# Bacteriophages specific to Shiga toxin-producing *Escherichia coli* exist in goat feces and associated environments on an organic produce farm in Northern California, USA

**DOI:** 10.1371/journal.pone.0234438

**Published:** 2020-06-11

**Authors:** Marion Lennon, Yen-Te Liao, Alexandra Salvador, Carol R. Lauzon, Vivian C. H. Wu

**Affiliations:** 1 Produce Safety and Microbiology Research Unit, U.S. Department of Agriculture, Agricultural Research Service, Western Regional Research Center, Albany, California, United States of America; 2 Department of Biological Sciences, California State University East Bay, Hayward, California, United States of America; Cornell University, UNITED STATES

## Abstract

Shiga toxin-producing *Escherichia coli* (STECs) contamination of produce, as a result of contact with ruminant fecal material, has been associated with serious foodborne illness. Bacteriophages (phages) that infect STECs have primarily been reported to be of cattle origin. However, they likely exist in other environments or in animals that share habitats with cattle, such as goats. To explore the presence and diversity of phages specific to STEC O157 and the top six non-O157 STECs in goat-associated environments, environmental samples consisting of feces (goat and cattle) and soil samples were collected monthly for six months from an organic produce farm. A variety of phages belonging to the *Myoviridae*, *Siphoviridae*, and *Podoviridae* families were isolated from all goat fecal and half of the soil samples. The most commonly isolated phages belonged to *Myoviridae* and were lytic against STEC O103. The isolated phages had different host ranges, but collectively, showed lytic activity against O157 and the top six non-O157 STEC strains excluding O121. Two non-O157 STECs (O174: H21 and O-antigen-negative: H18) were isolated from soil and cattle feces, respectively. Although prior studies have reported that goats shed STEC into the environment, the findings of the current study suggest that goat feces may also contain lytic STEC-specific phages. The phages of goat origin have the capacity to infect STECs implicated in causing foodborne outbreaks, making them potential candidates for biocontrol pending additional characterization steps. Further work is needed to determine if the addition of goats to the farm environment could potentially reduce the presence of STECs.

## Introduction

STECs comprise an important group of bacterial pathogens, which can cause diarrhea, hemorrhagic colitis, and occasionally life-threatening Hemolytic Uremic Syndrome (HUS) in humans. *E*. *coli* O157:H7 is the most notable STEC serotype, but 64% of the estimated 265,000 annual STEC infections in the United States are caused by the top 6 non-O157 serotypes, including O26, O45, O103, O111, O145, and O121 [1]. STEC pathogenicity is due to the production of Shiga toxins, encoded by *stx*1 or *stx*2, in combination with additional acquired virulence features [2]. During 1998 and 2017, the primary source of STEC infections is associated with the consumption of contaminated undercooked beef [3]. However, in recent years there are increasing outbreaks linked to produce because fresh produce is typically consumed raw with minimal treatment and processing [4,5].

Ruminants, such as cattle and goats, asymptomatically carry STEC in their digestive tracts [6], and their feces are the primary sources of STEC contamination on produce and in the environment [7–9]. Irrigation water sources and produce-growing soils are exposed to ruminant feces in agricultural settings inadvertently if STECs leach from ruminant enclosures through the soil, or via direct application of improperly treated animal manure [10,11]. Fresh produce that contacts contaminated irrigation water or soil can serve as a vehicle for transmission of STEC to humans [11]. In addition, livestock manure is frequently used for amending soils by the agricultural industry in the U.S. and abroad [10]. Organic farming operations, continually on the rise in the U.S. [12], must rely on the application of fresh or composted animal manure or plant debris alone for amending soil because the application of synthetic fertilizers does not comply with organic standards [13–15]. These practices may pose a higher risk of contamination by foodborne pathogens since ruminant feces is the primary reservoir of STEC [13].

Phages are the most abundant organisms on earth and the natural predators of bacteria [16,17]. Ruminant digestive tracts are also a reservoir for the phages specific to enteric microorganisms, and the general phage population in the rumen is reportedly greater than 10^7^ particles per milliliter [16]. Phages require access to specific host bacteria in order to replicate. Therefore, the existence of phages in an environment indicates the possible existence of its host within the same environment [18]. The phages specific for STEC are frequently isolated from cattle-associated environments [19–24], yet goats that co-mingle with cattle could possibly be an additional and nondisclosed source. To our knowledge, no studies exist which document the presence or successful isolation of phages specific to STEC from goats or goat-associated environments. Therefore, the objective of this research was to evaluate the presence of phages specific for STEC O157 and the top six non-O157 STECs in goat and cattle feces from animals that inhabit similar or nearby spaces at an organic produce farm.

## Materials and methods

### Farm site and sample collection

This field study did not involve contact with live animals, and no animals were harmed by any author. The collection of goat and cattle manure from the ground was permitted by the owners of the ranch located in Pescadero, California.

A small organic farm located in San Mateo County, CA, USA housed a consistently cohabiting herd of goats and one cow (10:1 ratio). The ruminants' shared space consisted of a barn situated in a 4,500 m^2^ pasture with continuous daily access to grass and browse. The ruminants were rotated to different fenced pastures on the property every few weeks for pasture management and to provide additional browsing opportunities. To survey for and isolate STEC-specific phages, goat and cattle feces and surrounding soil samples (400 grams each) were collected monthly for six sampling periods, from December 2017 to May 2018 (soil samples were collected for five months due to a sampling error on one sample date, total n = 17). For each sampling period, one cow fecal sample, one soil sample from within the browsing area but not directly underneath a fecal sample, and one composite goat fecal sample comprised of feces from multiple goats were collected into sterile Whirl-pak^®^ (Nasco, Fort Atkinson, WI) containers. The fecal samples were collected directly from the ground using sterile gloves. A sterile spade was used to collect soil 5 cm below the soil surface and did not include surface soil. The collection of multiple goats' feces was necessary to obtain the 400 g of the required sample since the moisture content of goat feces is lower than cattle feces. Samples were transported to the lab on ice and stored at 4°C until processing.

### Phage isolation and characterization

#### Bacterial host strains

Fourteen pathogenic STEC strains were obtained from the culture collection of the Produce Safety and Microbiology (PSM) Research Unit at the U.S. Department of Agriculture (USDA), Agricultural Research Service (ARS), Western Regional Research Center for isolating phages and determining host range (S1 Table).

#### Phage isolation and purification

A cocktail of STEC strains, used for phage isolation, was prepared by combining 1 ml of each of 14 individually enriched overnight cultures in Tryptic Soy Broth (TSB; Difco, Becton Dickinson, Sparks, MD). For enrichment, 15 g of fecal or soil sample, 35 ml sterile TSB, and 10 mM calcium chloride (CaCl_2_; VWR International, Radnor, PA), were homogenized using a Pulsifier (Microgen, Surrey, UK) for 15 s in a Whirl-pak^®^ bag. The bags were subsequently inoculated with 1 ml of the STEC cocktail and incubated at 37°C and 90 rpm for 24 h. The enrichments were subsequently centrifuged at 8,000 ×g for 10 min to pellet the host bacteria. The retained supernatants were filter-sterilized using a 0.22 μm filter (Millipore Sigma, Burlington, MA) to eliminate residual bacteria.

Spot tests were performed to identify the presence of STEC-specific phages in the enriched samples as previously described [25]. Briefly, sterile Tryptic Soy Agar (TSA; Difco, Becton Dickinson, Sparks, MD) plates were overlaid with molten TSA spiked with 200 μl of each singular STEC strain that was used to enrich with the environmental samples (S1 Table). Six microliters of each phage lysate were aliquoted onto the strain-inoculated TSA plates and incubated at 37°C for 24 h. The presence of phage was identified by a zone of clearing within the confluent bacterial growth (a plaque), indicating the presence of phages lytic against the tested STEC strain. Three spots from each plaque were picked randomly by pipette and combined in 1 ml of 0.1% peptone water and vortexed to homogenize for 2 min to gently free the phages from the agar. Plaque suspensions were then diluted 10-fold to 10^−7^, followed by a double-layer plaque assay with the primary strain of isolation to enumerate the phages as previously described [25]. The primary strain of isolation was the strain used in the spot test assay with the clearing zone on the bacterial lawn caused by phage lysis. After incubation of the plates at 37°C for 24, a plaque was picked from the plaque-assay plate and then subjected to double-layer plaque assay with the method as described above, and three passages of single-plaque purification were conducted to purify the phages.

The purified phages were propagated by mixing 40 ml TSB supplemented with 10 mM CaCl_2_, 200 μl of an overnight culture of the primary strain of isolation, and 50 μl purified phage suspension, and incubated at 37°C and 90 rpm for 24 h. The phage propagates were centrifuged at 8,000 ×g for 10 min to pellet the host bacteria and the retained lysates were filter-sterilized individually using a 0.22 μm filter (Millipore Sigma, Burlington, MA). The lysates were diluted serially via 10-fold dilutions based on prior plaque counts and a final titer assessed via the double-layer plaque assay. The resulting phage lysate was concentrated to 500 μl using an Amicon 100 kDa filter tube (Millipore Sigma, Burlington, MA), treated with 100 U/ml DNase I (Zymo Research, Tustin, CA) per manufacturer's instructions, and eluted in 500 μl ultrapure water.

The concentrated phage lysates were then purified via cesium chloride (CsCl) gradient ultracentrifugation in an Optima MAX-XP Ultracentrifuge (Beckman Coulter, Brea, CA) [26]. Briefly, three densities of CsCl: Rho 1.7, Rho 1.5, and Rho 1.3 were reconstituted in 10% Saline Magnesium (S.M.) buffer (Teknova, Hollister, CA) and filter sterilized (0.2 μm), then layered in 5 ml ultracentrifuge tubes (Beckman Coulter, Brea, CA) with the highest density solution on the bottom. Individual phage lysates were gently pipetted on top of the CsCl solution and centrifuged at 131,300 ×g for 24 h at 4°C. Viral particle retentates at the expected location between the Rho 1.7 and Rho 1.5 layers were retained [27,28]. CsCl-treated phages were dialyzed in a 20 kDa MWCO Slide-A-Lyzer™ Dialysis Cassette (Thermo Fisher Scientific, Waltham, MA) per manufacturer's instructions. The dialysis procedure with modifications was used [29]. Briefly, a more conservative approach was taken, including a shorter dialysis time for the initial dialysis step in 1 M NaCl and an additional step of dialysis in 0.5 M NaCl prior to dialysis against 100% S.M. buffer, followed by 10% S.M. buffer. The weaker final S.M. buffer step was chosen in order to reduce any potential impact of S.M. buffer on downstream application steps.

#### Phage host range determination

Phages were assessed for the host range via the spot test as described in the previous section against the original STEC hosts (S1 Table), two isolated STECs, *E*. *coli* O174:H21, O-:H18, in the current study, *B*. *subtilis* ATCC 11778, *Pseudomonas aeruginosa* RM 3291 (USDA ARS), *Salmonella* Typhimurium ATCC 14028, and *Staphylococcus aureus* ATCC 25923.

#### Transmission electron microscopy

Phage morphology was investigated using a FEI Tecnai G2 electron microscope as previously described [25]. Briefly, a 6 μl aliquot of Amicon-concentrated, CsCl-purified phage lysate was applied to a copper mesh PLECO grid (Ted Pella Inc., Redding, CA), blotted with 90 mm Whatmann® filter paper Grade 597, negative-stained for 10 s with 8 μl 0.75% uranyl acetate (Sigma-Aldrich, Darmstadt, Germany), and blotted again. The grids were dried for 2 h and stored at ambient temperature for viewing within 24 h. Grids were evaluated at magnifications ranging from 38,000–40,000 × for 20 nm scale images, and 20,000 × magnification for 50 nm scale images.

#### Phage DNA extraction and *stx* gene screening

The detection of *stx* genes within the isolated phages was investigated as previously described with minor modifications [25]. DNA was extracted from phages using a Phage DNA Isolation Kit per manufacturer's instructions (Norgen Biotek Corp., Ontario, Canada). Twenty five microliters of reaction volume, composed of 10 μM *stx*1 or *stx*2 forward/reverse primer pairs

([5'-CATCGCGAGTTGCCAGAATG-3’/5’-AATTGCCCCCAGAGTGGATG-3’] or [5'- GTATACGATGACGCCGGGAG-3'/ 5'- TTCTCCCCACTCTGACACCA-3'], respectively), 12.5 μl 2X Promega Master mix (Promega Corp., Madison, WI), nuclease-free water, and 1 μl DNA template. The parameters of thermocycle step for denaturing, annealing, and extension temperatures were 95°C, 56°C and 72°C, respectively for *stx*1 gene, and 95°C, 58.1°C and 72°C for *stx*2. PCR amplification of 28 cycles were used. The resulting PCR product was electrophoresed in a 1.5% gel containing 1X Gel Red (Biotium, Fremont, CA) at 120 V for 1.5 h and imaged with an Alpha Imager U.V. gel box (Alpha Innotech, San Leandro, CA). Expected PCR product targets were 119 bp (*stx*1) and 104 bp (*stx*2). *E*. *coli* O157 Strain RM1484 (USDA ARS WRRC) was used for a positive control because it contains both *stx*1 and *stx*2.

#### Phage genome size assessment and Restriction Fragment Length Polymorphism (RFLP) profile

The genome size of the 14 isolated phages was examined by Pulsed Field Gel Electrophoresis (PFGE), as previously described [30,31]. Two hundred microliters of whole phage particles were solidified in 1.2% SeaKem® Gold Agarose plugs to prevent shearing of DNA and incubated for 2 h with phage lysis buffer and 20 mg/ml Proteinase K (New England Biolabs, Ipswich, MA). A plug containing purified O121-specific phage with 134 kb genome size was prepared and served as a positive control. After washing, plugs were embedded on the PFGE comb in a 1% SeaKem® Gold Agarose gel and run with 0.5 M tris-borate-EDTA buffer at 14°C and 6 V/cm with pulse times of 2.2–54.2 s for 20.5 h using the Chef DR-II Mapper electrophoresis system (Bio-Rad, Hercules, CA). *Salmonella* Branderup H9812 (Bio-Rad Laboratories, Hercules, CA) digested with the restriction enzyme *XbaІ* (New England Biolabs, Ipswich, MA) was chosen for a ladder because it is used as a global reference standard by PulseNet, the CDC's National Laboratory Network Database [32]. After digestion with *XbaІ* for 3 h, the *Salmonella* Branderup H9812 genome resulted in bands ranging from 20.5 to 1,135 kb in electrophoresis [33]. Gels were stained with 3X Gel red (Biotium, Fremont, CA) for 20 min, destained in ultrapure water, and then imaged with an Alpha Imager U.V. gel box (Alpha Innotech, San Leandro, CA). Bands of phage at their respective bp size were cut from the gel over U.V. illumination, and *stx* presence was assessed for each after extracting DNA with the QIAquick Gel Extraction Kit (Qiagen, Hilden, Germany).

RFLP profiles of select broad host range phages were examined using PFGE, as described previously [31]. Plugs of 1.2% SeaKem® Gold Agarose of phage isolates were digested separately with both restriction enzyme of *Eco*RV and *Hin*dIII for 16 h at 37°C and then electrophoresed with a 1 kb Plus ladder (Sigma-Aldrich, St. Louis, MO) for 5 h at 14°C and 6 V/cm with pulse times of 1.0–42.4 s. Gels were stained with 3X Gel red (Biotium, Fremont, CA) for 20 m, destained in ultrapure water, and then imaged with an Alpha Imager U.V. gel box (Alpha Innotech, San Leandro, CA).

### Environmental STEC isolation

STEC isolation was performed following a modified protocol described previously [34]. Briefly, to isolate STEC from the same samples as STEC-specific phages, 10 g of fecal or soil sample and 90 ml of TSB were homogenized via Pulsifier (Microgen, Surrey, UK) for 15 s in a Whirl-pak^®^ bag and incubated at 37°C and 90 rpm for 24 h. Enrichments were centrifuged at 8,000 xg for 10 min and pellets retained. An aliquot of 50μl of enrichment sample was transferred to 0.2 ml PCR tubes, and DNA was extracted via boiling at 100°C for 20 m in a Bio-Rad C1000 Touch Thermal Cycler (Bio-Rad, Hercules, CA) followed by 5,000 xg centrifugation for 5 m to retain DNA in the supernatant. Due to the variable behavior of environmentally-isolated bacteria and the potential for suboptimal or stressful enrichment conditions to prevent their culture or impact their viability, each enrichment was assessed for the presence of STEC or *stx* via three methods: traditional PCR and gel electrophoresis, RT-PCR, and plating on selective media. DNA extracted via heat lysis was subjected to traditional PCR and gel electrophoresis per same method and conditions as the phages (see *Phage DNA extraction and* *stx* *gene screening)*. DNA extracted via heat lysis was subjected to RT-PCR for *stx*1, *stx*2abc, and *stx*2ex screening using primers and probes designed previously [34] with a BioRad CFX-96 Real-Time system (Bio-Rad, Hercules, CA). *E*. *coli* O157 Strain RM1484 (USDA ARS WRRC) was used for a positive control due to the presence of *stx*1, *stx*2abc, and *stx*2ex. The information of primer and probe sequences is presented in S2 Table.

Pelleted enrichments not subjected to DNA extraction were simultaneously plated on four selective media for isolation: Sorbitol MacConkey (Difco Labs, Detroit, MI) with cefixime (0.05μg/mL; Introgen/Dynal) and tellurite (2.5μg/mL; Introgen/Dynal), Rainbow Agar O157 (Biolog, Hayward, CA) containing novobiocin (20μg/mL; Sigma-Aldrich) and tellurite (0.8μg/mL; Introgen/Dynal) (NT-RA), CHROMagar O157 (DRG International, Mountainside, New Jersey), and modified Sheep Blood Agar (mSBA) consisting of defibrinated sheep's blood (BioMerieux, Durham, NC), BBL Blood Agar Base (Becton Dickinson, Sparks, MD), 10mM CaCl_2_, 0.5mg/L mitomycin C (Thermo Fisher Scientific, Waltham, MA), and 50 mg/L X-Gal (Teknova, Hollister, CA). The colonies with positive results as specified by the media manufacturers were subjected to *stx* screening.

Isolates deemed presumptively positive for STEC were then inoculated in 10 ml TSB and incubated for 24 h at 37°C. Bacterial cells were centrifuged to a pellet and then plated on CHROMagar STEC (DRG International Inc., Springfield, NJ). Mauve isolates were considered presumptive positive and were individually inoculated in 10 ml TSB and incubated for 24 h at 37°C. Bacterial cells were centrifuged to a pellet, and DNA was extracted as described above. RT-PCR or traditional PCR and gel electrophoresis was used to screen for *stx* genes as described above.

#### STEC serotyping

Isolates resulting in Ct values of <20 from RT-PCR, bands at expected bp during *stx* screening by traditional PCR and gel electrophoresis, or visual indication of STEC on selective and differential media were serotyped using the Luminex Molecular STEC Serotyping kit on the MagPix Multiplexing System (Luminex, Austin, TX) [35]. Briefly, DNA of presumptive isolates was subjected to PCR in a 25 μl reaction volume with PCR Master Mix (Promega Corp., Madison, WI) and STEC primer mix for amplifying the O antigen of *E*. *coli* serogroups O26, O45, O91, O103, O104, O111, O113, O121, O128, O145, and O157, as well as *eae* and *agg*R genes. The information of primer and probe sequences is presented in S3 Table. Thermocycle parameters for denaturing, annealing, and extension temperatures were 94°C, 52°C, and 72°C, respectively. PCR amplification of 40 cycles was used.

Following PCR, the amplified DNA from individual isolates was hybridized to MagPlex beads for 15 min at 94°C and 30 min at 52°C. After beads were separated using a heated magnetic plate, a reporter probe was added to each and incubated for 10 min, followed by reading of fluorescence via the MagPix system. Three positive control cocktails consisting of the Top 7 O-serogroups (O157 and Top 6 non-O157) were validated and run with every assay. For data analysis, the signal-to-noise ratio for each analyte, or amplified DNA of presumptive isolates bound to a specific region of the MagPlex bead and bearing a reporter, was calculated against the background noise by dividing the Mean Fluorescent Intensity (MFI) of the analyte by the MFI of a nuclease-free H_2_O sample. Analytes with a signal-to-noise ratio of >5.0 were considered to be positive for that analyte. STEC strains unable to be serotyped using the Luminex Molecular STEC Serotyping kit were sent to the *E*. *coli* Reference Center (Pennsylvania State University, University Park, PA) for serotyping and assessment of virulence genes including *stx* and *eae*.

## Results

### STEC-specific phage isolation and assessment of diversity

#### The presence and host range of the STEC-specific phages

Approximately 59% of the samples (n = 17) collected were positive for the presence of STEC-specific phages. STEC-specific phages were found in all (6/6) composite goat fecal samples (n = 6), 60% of the soil samples (n = 5), and 18% of cow fecal samples (n = 6). After the isolation and purification process, the results showed the presence of 14 phages lytic against various STEC strains, designated P1 through P14 (Table 1).

**Table 1:** Isolation of STEC-specific phage and STEC from different environmental sources and sampling periods.

Sampling Period	Source					
	Goats		Cow		Soil	
	Phage	STEC	Phage	STEC	Phage	STEC
1	P1, P2	-	-	-	-	-
2	P3, P4	-	-	-	-	-
3	P5	-	-	O-:H18	P6, P7	O174:H21
4	P8, P9	-	-	-	*	*
5	P10	-	-	-	P11	-
6	P12	-	P13	-	P14	-

- indicates no STEC strain was isolated.

*indicates source was not sampled.

Host range assay via the spot test showed that 10 phages (71%), isolated from 8 samples (5 goat, 2 soil, and 1 cattle), were presumptive lytic against STEC O103 (Table 2). Phages with the ability to infect more than 4 STEC strains were arbitrarily designated to have a broad host range [36]. Nine isolated phages (64%) possessed a broad host range, all of which belonged to the family *Myoviridae* and showed lytic activity against O103 and the two isolated STECs, O174: H21 and O-untypable: H18 isolated from soil and cattle feces, respectively. The isolated phages were considered presumptively lytic.

**Table 2:** Host range of the 14 isolated phages against various STEC, 1 *Bacillus*, 1 *Pseudomonas*, 2 *Salmonella*, and 2 non-O157 STEC strains- O174 and O, isolated in the current study, by the spot test.

	Phage ID													
Strain	P1	P2	P3	P4	P5	P6	P7	P8	P9	P10	P11	P12	P13	P14
*E*. *coli* O103:H2	++	-	++	-	++	-	-	H	++	++	++	-	+	++
*E*. *coli* O103:H-	H	-	H	++	++	-	-	++	H	H	H	-	H	H
*E*. *coli* O111:H-	+	-	+	H	H	-	-	-	+	+	+	-	-	-
*E*. *coli* O111:H-	+	-	+	-	++	-	-	-	+	-	-	-	-	-
*E*. *coli* O121:H19	-	-	-	-	-	-	-	-	-	-	-	-	-	-
*E*. *coli* O121:H-	-	-	-	-	-	-	-	-	-	-	-	-	-	-
*E*. *coli* O145:H-	-	-	-	-	-	H	++	-	-	-	-	H	-	-
*E*. *coli* O145:H-	-	-	-	-	-	++	H	-	-	-	-	+	-	-
*E*. *coli* O157:H7	+	-	-	-	+	-	-	-	-	-	-	-	-	-
*E*. *coli* O157:H7	+	-	-	-	+	-	-	-	-	-	-	-	-	-
*E*. *coli* O26:H-	+	-	+	+	++	-	-	-	+	+	+	-	-	-
*E*. *coli* O26:H-	+	-	++	-	++	-	-	-	+	+	+	+	-	-
*E*. *coli* O45:H-	-	++	-	-	-	-	-	-	-	-	-	-	-	-
*E*. *coli* O45:H-	-	H	-	-	-	-	-	-	-	-	-	-	-	-
*E*. *coli* O174:H21	++	-	++	+	++	-	-	++	++	++	++	-	-	++
*E*. *coli* O-:H18	++	-	++	+	++	-	-	++	++	++	++	-	-	++
*B*. *subtilis* ATCC 11778	-	-	-	-	-	-	-	-	-	-	-	-	-	-
*P*. *aeruginosa* RM 3291	-	-	-	-	-	-	-	-	-	-	-	-	-	-
*S*. Typhimurium ATCC 14028	-	-	-	-	-	-	-	-	-	-	-	-	-	-
*S*. *aureus* ATCC 25923	-	-	-	-	-	-	-	-	-	-	-	-	-	-

"H" indicates lysis and the primary host strain used for phage isolation and purification. Degree of lysis: ++ indicates strong lysis; + indicates presence of lysis;—indicates no lysis.

#### Phage morphology

**Fig 1:**
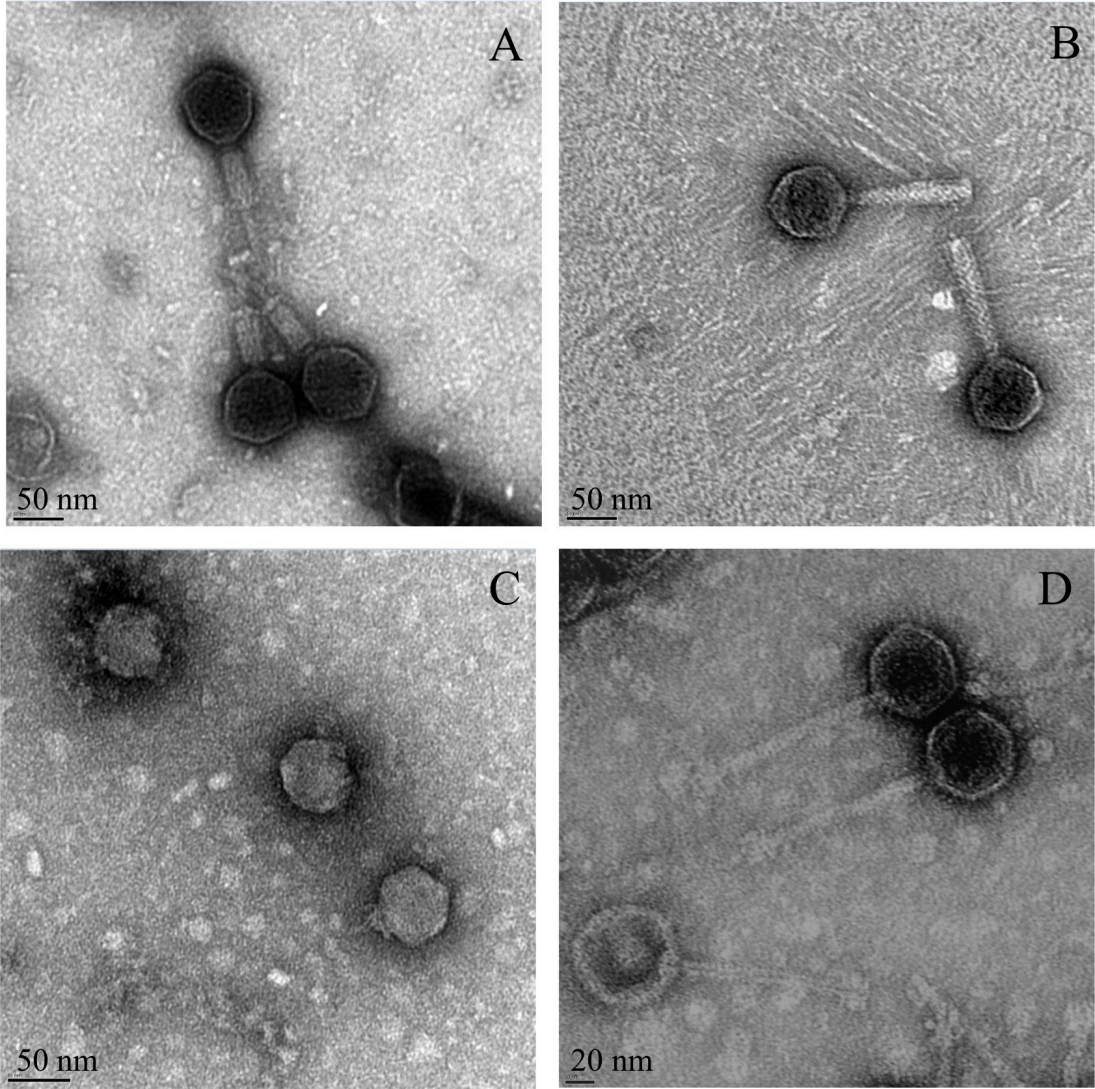
Morphology of isolated STEC-specific phages. The morphology of phage P1, belonging to the family *Myoviridae*, isolated from goat feces with tails in a contractile state (A); the morphology phage P3, belonging to the family *Myoviridae*, isolated from goat feces (B); the morphology of phage P2, belonging to the family *Podoviridae*, isolated from goat feces (C); the morphology of phage P7, belonging to the family *Siphoviridae*, isolated from soil (D).

All phages capable of infecting STECs belong to the order *Caudovirales*, and observation by Transmission Electron Microscopy (TEM) revealed phage morphologies similar to those in the families *Myoviridae* (7 of 10 isolates), *Siphoviridae* (2 of 10 isolates), and *Podoviridae* (1 of 10 isolates) (Fig 1, Table 3).

**Table 3:** The genetic and morphological characterization of the 14 isolated phages.

Characterization	Phage ID													
	P1	P2	P3	P4	P5	P6	P7	P8	P9	P10	P11	P12	P13	P14
Morphology^α^	M	P	M	*	*	S	S	M	M	M	M	*	*	M
Genome size est. (kb)	80	30	80	80	80	35	35	80	80	80	80	33	80	80

^α^Phage morphologies include the families of (M) *Myoviridae*, (P) *Podoviridae*, and (S) *Siphoviridae*

*indicates no phage morphology was obtained.

All phages were negative of *stx* genes.

#### Molecular features of the phages (*stx* screening, genome size & RFLP)

All the isolated phages were negative of the virulence genes of *stx1* and *stx2*; therefore, together with the host range test, these phages might have the potential to mitigate the target STEC strains.

PFGE was used to estimate genome size of the isolated phages (Table 3, S1 Fig). The positive control phage resulted in 2 bands, at 134 kb and 120 kb. The *Myoviridae* phages, primarily lytic for O103 and O111, possessed a genome size of ~80 kb. The *Siphoviridae* phage genomes resulted in approximately 35 kb in size, and the *Podoviridae* possessed an approximate genome size of ~30 kb. Phages unable to be examined by TEM possessed genome sizes of 80 kb (P4, P5, and P13) and 33 kb (P12).

RFLP profiling was used to differentiate between *Myoviridae* isolates that could not be distinguished using the host range, morphological observation, and estimated genome size alone. P4 and P13 were not assessed for RFLP profiling because their host range results were different from the other O103 and O111 specific phages. Specifically, P4 showed lytic activity against only one out of two of the O103 and O111 strains tested. P13 only showed lytic activity against O103. Phage DNA was not susceptible to cleavage by *Hind*III, but *Eco*RV digestion revealed that isolate P5, possessing the broadest host range of all isolated phages and O111 as the primary strain of isolation, differed from the profiles of other O103-specific myophages with a broad host range (P3, P8, P10, and P11) by one band at approximately 2 kb (S2 Fig). Isolate P5 resulted in 15 bands, whereas isolates P3, P8, P10, and P11 resulted in 16 bands.

### STEC strain isolation and serotyping

All the samples assessed for phages were also tested for the presence of STECs using culture and PCR-based methods. No STEC O157 and top 6 non-O157 were found in the animal samples regardless of small sample size and grazing conditions (Table 1). Non-culture-based assay of goat feces collected during the 1^st^ sampling period resulted positive for the presence of *stx*1 and soil collected during the 3^rd^ sampling period resulted positive for the presence of *stx*2. All 17 enrichments were also assessed for *stx* genes via RT-PCR and were plated on selective and differential media. The use of four selective and differential media (mSBA, Rainbow Agar O157, CHROMagar O157, and CT-SMAC) resulted in 139 isolates needing further assessment for *stx* genes. Each of the 139 isolates was plated on CHROMagar STEC, and the presumptive isolates of STEC were assessed for *stx* genes via RT-PCR. If the strains were positive of *stx* genes, the bacterial isolates were further serotyped using the Luminex MagPix STEC Molecular Serotyping Kit.

The *stx*1-positive goat fecal sample was subsequently plated on selective and differential media but a *stx*1-positive STEC did not grow on either selective or nonselective media. An isolate from soil from the 3^rd^ sampling period was successfully cultured on selective and differential media but could not be serotyped using the Luminex kit (S4 Table and S5 Table). Additionally, plating of the cattle fecal enrichment from the 3^rd^ sampling on selective and differential media resulted in the culture of a *stx2*-positive STEC and was the only isolate observed as a mauve colony/positive for both CHROMagars. Both *stx*2-positive isolates were sent to the Penn State *E*. *coli* Reference Center for serotyping and virulence assessment. The *stx*2-positive soil sample isolate was serotyped as O174: H21 and the *stx*2-positive cattle feces isolate did not match any known O-serogroups, but the flagellar antigen was determined to be H18. Both isolates lacked the *eae* gene, which encodes for intimin.

## Discussion

The prevalence of STEC-infecting phages in ruminants has been extensively studied in cattle [19–24]. In this study, various STEC-specific phages were isolated from goat-associated environments, which showed lytic capacity against more than one serogroup of STEC strains. The current findings are consistent with the results from a previous study in which cattle feces harbored diverse STEC-infecting phages capable of lysing more than one clinically important serogroup [23]. Phages with lytic activity against greater than four strains were arbitrarily designated as having a broad host range. In this study, 9 of the 14 isolated phages resulted in a broad host range, showing strong lytic activity against *E*. *coli* O103, O26, O111, O174, and the O-untypable strains. Additionally, the phages deriving from the goat feces possessed the broadest host ranges. Other studies have speculated that phages with a broad host range have a clear advantage over phages with a narrow host range in terms of encountering more susceptible host cells [37]. Since the presence of phage in an environment depends on the presence of a host in the same environment [38], phages with a narrow host range may have limited propagation into the environment [39]. Therefore, phages with broad host range, such as those we found, are more likely to be prolific in a goat-cattle environment and more likely to be isolated. None of the phages that we found were lytic against *Salmonella* Typhimurium or *Pseudomonas aeruginosa*, or two Gram-positive strains, *Bacillus subtilis* and *Staphylococcus aureus* (Table 2), typically found in similar environments that act as opportunistic pathogens. The phenomenon is likely due to the differences of host cell receptors between Gram-negative and Gram-positive bacteria [40].

In this study, the majority of isolated phages belonged to the *Myoviridae* family, even though phages typical of all three families of the order *Caudovirales* were isolated. The myophages were predominantly isolated from goat feces and possessed the broadest host ranges. A previous study reported that phages belonging to family *Myoviridae* were the most frequently isolated phage morphology from cattle feces [23]. Additionally, a review article also demonstrated that among more than 60 reported phages isolated from various environmental sources that were lytic against STEC strains, the morphologies of most phages belong to the *Myoviridae* family [41]. On the other hand, the results of the current study show that the isolated phages belonging to the families *Podoviridae* and *Siphoviridae* demonstrated a narrow host range infecting only one STEC serogroup (O45 and O145, respectively). Hallewell *et al*. similarly found that the phages belonging to *Myoviridae* exhibited a broader host range than those of family *Siphoviridae* [20]. Myophages reportedly have a broader host range in comparison to siphophages or podophages due to their more sophisticated baseplates [40]. The morphologies of some phages, such as P4, P12, and P13 in this study, could not be obtained under TEM because the phages either did not maintain viability in storage or had an insufficient titer level [42]. Additionally, low phage titers of the isolated phages with a strong spot test results were also observed, which could be due to the effects of "lysis from without"; the lysis of bacterial hosts occur because of excessive phage adsorption and initiation of replication, but no phage progenies are produced [43]. Alternatively, since the phages are considered presumptively lytic until further characterization steps are performed, the phage could be temperate or lysogenic [44]. Overall, the findings of the current study indicate that morphologically-diverse STEC-specific phages potentially exhibiting multiple life cycles exist in the goat-associated environments we surveyed.

PFGE results of the isolated phages revealed estimated genome sizes ranging 30–80 kb. Phages with a broader host range had an estimated 80 kb genome size and those with a narrower host range had relatively smaller genome sizes ranging from 30–35 kb, except for phage P13 (Table 3). Furthermore, phages with 80 kb, 35 kb, and 30 kb estimated genome sizes belonged to the families *Myoviridae*, *Siphoviridae*, and *Podoviridae*, respectively. A previous study isolated four siphophages, lytic against *E*. *coli* O157:H7, from cattle feces with estimated genome sizes of 44 kb [31]. Hallewell *et al*. isolated several lytic phages specific to *E*. *coli* O157:H7 from cattle feces and found that the estimated genome sizes for the phages belonged to the families *Siphoviridae* and *Myoviridae* were 42.4 kb and 86.1 to 182.7 kb, respectively [22]. Wang *et al*. found a variety of phages from the animal origin that had lytic activity against non-O157 STEC and belonged to the families of *Myoviridae*, *Siphoviridae*, and *Podoviridae* with estimated genome sizes of 68–177 kb, 126 kb, and 38 kb, respectively [23]. In the current study, the genome sizes estimated are similar to the prior published genome sizes of the STEC-specific phages.

PFGE and RFLP profiling were subsequently used together to further differentiate between the phages with the most similar host ranges. The results revealed that phage P5 resulted in one less band at approximately two kb in comparison to other myophages with a broad host range—P3, P8, P10, and P11. The band profile of phage P5 might indicate a difference in genes coding for phage tail structures in charge of binding the receptors on the host bacterial membrane [23]. This aligns with the fact that the primary strain of isolation for P5 was *E*. *coli* O111 versus the primary strain of isolation for phages P3, P8, P10, and P11 was *E*. *coli* O103 (Table 2). In a prior study, Niu *et al*. assessed restriction profiles of four *E*. *coli* O157-specific siphophages isolated from cattle feces [31]. The authors found that the digestion profiles of these phages differed slightly and correlated with differences in host range despite identical estimated genome size. Wang *et al*. also assessed RFLP profiles of tailed phages isolated from cattle feces and noted that of the 10 restriction enzymes tested, only *Eco*RV was capable of digesting T4 phages, which belong to the family *Myoviridae*, due to the presence of glycosylated hydroxylmethyl cytosine instead of cytosine [23]. The study conducted by Hallewell *et al*. also observed the same finding [22]. Thus, in the current study, it is possible that since *Eco*RV digested the DNA of P5 and P9 but was not digested by *Hind*III, this is a likely indication that these phages may be similar to the T4 species of the *Myoviridae* family. These findings are aligned with our results in that the isolated phages with the same morphology, similar host range, and estimated genome size exhibited minor variations in the genomic profile.

No Top 7 STECs were isolated from any sample collected during the 6-month sampling period; yet, collectively, the isolated phages showed broad diversity and infectivity for resident STECs and all Top 7 STECs excluding O121. Although cattle with pasture access tend to have higher STEC frequencies [45], the extensive pasture size, limited ruminant population, and subsequent low animal density of the current study sampling area may have contributed to the overall limited isolation of STEC [46]. Additionally, STECs could have been present but were uncultivable. There are many causes for cells to resist culture, including cell injury or stress, or unsatisfactory simulation of the organism's optimal environmental conditions [47]. The *stx*-1 positive goat fecal sample from PCR did not yield a culture; this might be because an initial enrichment step was needed or the detected *stx* genes might derive from another bacterial genus [48]. However, the methodology used here allowed for the isolation of two non-top 7 STECs: *E*. *coli* O174: H21 and *E*. *coli* O-untypable: H18 strains were isolated from a soil sample and cattle feces, respectively (Table 1). Since the methodology was sufficient to detect STECs, and STECs were detected at this farm, it is then possible that the resident phages impacted the presence of colocalized STECs. Pausz *et al*. found that the diversity of a bacterial community in an environment was at least as diverse as the phage community [49]. Switt *et al*. isolated phages capable of lysing the most common *Salmonella* serovars from dairy farms with a prior history of *Salmonella* presence; however, the samples assessed for *Salmonella*-specific phages were not simultaneously tested for *Salmonella* presence. The authors concluded that *Salmonella*-specific phage diversity reflected host diversity on dairy farms and that phages may impact the ecology of their hosts within shared environments [36]. These prior studies indicated that the phages were present in the environment where their bacterial hosts existed. However, the results in the current study might not be able to claim a similar finding due to the small sample size and the lack of historical data on STEC prevalence.

An interesting finding from this study is that the samples from the goat-associated environments which contained phages simultaneously lacked the presence of suitable bacterial hosts they could infect. This phenomenon is corroborated by the previous studies that focused on the correlation between STEC-specific phages and STEC in cattle. For example, Niu *et al*. found a weak but significant negative correlation existed between the isolation of STEC-specific phages and the isolation of *E*. *coli* O157 in cattle fecal samples. The authors concluded that the likelihood of *E*. *coli* O157-shedding was reduced if cattle in the same pen harbored *E*. *coli* O157-specific phages [19]. Their findings suggest that phages act prophylactically, since phages isolated from ruminant-associated environments controlled for STEC in their immediate environment. Hallewell *et al*. found that cattle shedding low levels of *E*. *coli* O157 (< 10^4^ cfu/g) correlated with a higher prevalence of colocalized STEC-specific phages in comparison to "super shedders" and concluded that endemic phages may mitigate the presence of colocalized STECs in a feedlot environment [22]. Liao *et al*. found the presence of free STEC-specific phages likely conferred a mitigating effect on their STEC hosts in a pre-harvest produce environment [25]. Thus, these findings suggest that phages impact the shedding dynamics of STEC in numerous environments. The limited sample and population size in the current study prohibit claiming that the isolated phages impacted STEC presence. However, the current data indicate that some of the isolated phages show antimicrobial potential for STEC in nature because of the lack *stx* genes, strong lytic activity, and a broad host range against several clinically relevant STEC serogroups.

## Conclusions

To the best of our knowledge, this is the first study looking at the presence and diversity of STECs and associated phages in goat feces and the surrounding environments where goat feces are present in an organic produce-growing setting in the U.S. The data indicate that diverse STEC-specific phages can exist in goat feces and companion surrounding environments, and that these phages are capable of infecting resident STECs and STEC strains implicated in causing foodborne outbreaks. The results also suggest that goat-derived STEC-specific phages may mitigate the presence of STECs in the produce-growing environment since the samples which contained phages lacked bacterial hosts they could infect. However, feces may contain other components and molecules with bactericidal effect; thus further testing of fecal and soil samples are needed. Nevertheless, the findings of this study likely suggest that the presence of STEC-specific phages may adversely impact the presence of *E*. *coli* O157 and Top 6 non-O157 STEC strains in soil, and thus a possible control measure for STEC shedding. Further study is necessary to characterize the isolated phages to explore the biocontrol potential of STEC strains.

## Supporting information

S1 Fig PFGE image showing the estimated genome sizes of the 14 isolated phages. Phages P1-P7 **(a)** and P8-P14 **(b)**. The control sample, O121-specific phage, resulted in its previously sequenced genome size of 134 kb.
(DOCX)

S2 Fig PFGE image of *Eco*RV-digested phage DNA. Red arrow at 2 kb indicates a grouping of 2 bands for P5 versus 3 bands for P3, P8, P10, and P11. Phage Lambda DNA was treated with *Eco*RV to serve as a positive control and undigested Lambda phage DNA served as a negative control.
(DOCX)

S1 Table STEC strains used for isolating STEC-specific phages. Strains excluding O157-2 were obtained from USDA ARS WRRC. Strain O157-2 was obtained from the ATCC, American Type Culture Collection, Manassas, VA. Strain O121-1 was obtained from the CDC, Centers for Disease Control and Prevention, Atlanta, GA.
(DOCX)

S2 Table RT-PCR Primers and probes used for *stx* gene screening in phage or bacteria [34]. (DOCX)

S3 Table Luminex MagPix Molecular STEC Serotyping primers and probes [35]. (DOCX)

S4 Table Luminex MagPix Mean Fluorescent Intensity (MFI) Results of Soil-Isolated STEC. The Luminex MagPix CCD camera interrogates each bead and detects the amount of reporter/fluorescence bound to each bead region then calculates the mean of the fluorescence reads for that particular bead region.
(DOCX)

S5 Table Luminex MagPix Signal-to-Noise (S/N) Ratios of Soil-Isolated STEC. For data analysis, the S/N ratio for each analyte, or amplified DNA of presumptive isolates bound to a specific region of the MagPlex bead and bearing a reporter, was calculated against the background noise by dividing the mean fluorescent intensity (MFI) of the analyte by the MFI of the nuclease-free H2O sample. Analytes with a signal-to-noise ratio of >5.0 were considered to be positive for that analyte, however, due to the sensitive nature of the assay, any analytes resulting in lone signals of >1 for only one serogroup were subjected to serotype confirmation.
(DOCX)
