# Supplementary material for: Bacteriophages specific to Shiga toxin-producing Escherichia coli exist in goat feces and associated environments on an organic produce farm in Northern California, USA
Source: PLoS One. 2020 Jun 11;15(6):e0234438. doi: 10.1371/journal.pone.0234438 (PMC7289414; doi:10.1371/journal.pone.0234438)
Supplement: S1 Fig — Phages P1-P7 (a) and P8-P14 (b). The control sample, O121-specific phage, resulted in its previously sequenced genome size of 134 kb. (DOCX) [file pone.0234438.s001.docx]

**a)**


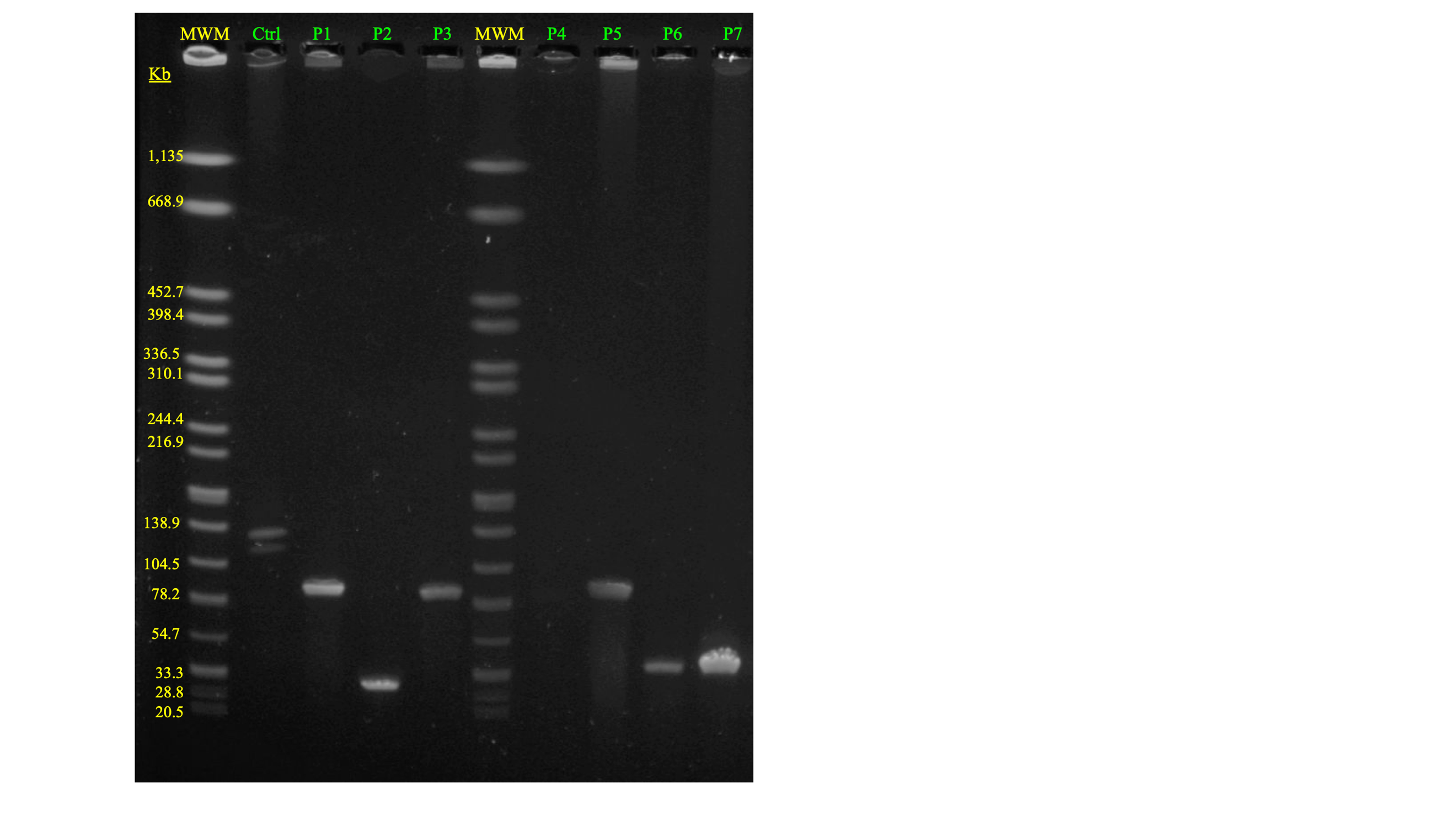


**b)**


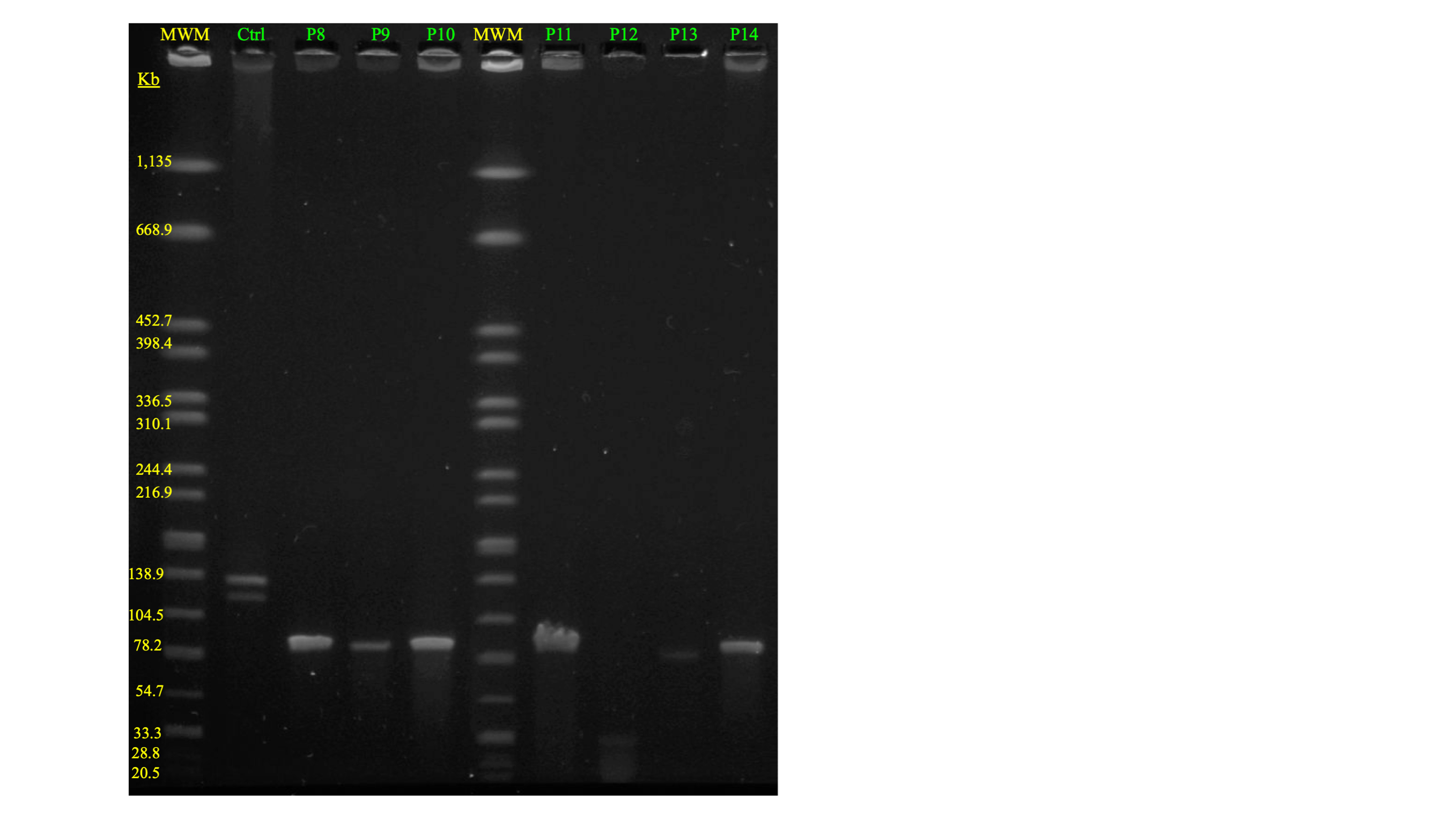


**Fig S1.** **PFGE image showing the estimated genome sizes of the 14 isolated phages**. Phages P1-P7 **(a)** and P8-P14 **(b)**. The control sample, O121-specific phage, resulted in its previously sequenced genome size of 134 kb.
