# Supplementary material for: Bacteriophages specific to Shiga toxin-producing Escherichia coli exist in goat feces and associated environments on an organic produce farm in Northern California, USA
Source: PLoS One. 2020 Jun 11;15(6):e0234438. doi: 10.1371/journal.pone.0234438 (PMC7289414; doi:10.1371/journal.pone.0234438)
Supplement: S2 Fig — Red arrow at 2 kb indicates a grouping of 2 bands for P5 versus 3 bands for P3, P8, P10, and P11. Phage Lambda DNA was treated with EcoRV to serve as a positive control and undigested Lambda phage DNA served as a negative control. (DOCX) [file pone.0234438.s002.docx]

*
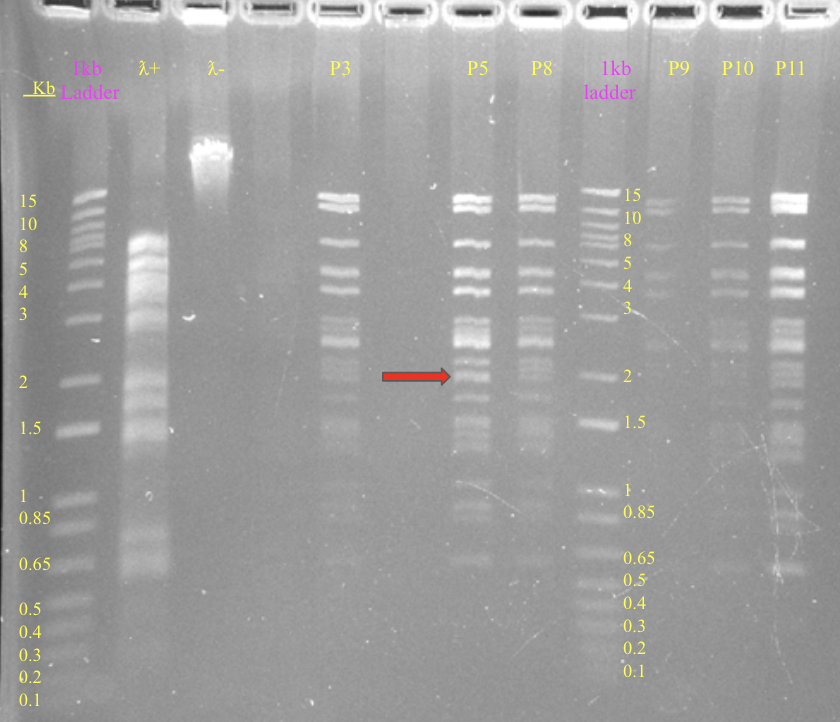
*

**Fig S2.** **PFGE image of *Eco*RV-digested phage DNA**. Red arrow at 2 kb indicates a grouping of 2 bands for P5 versus 3 bands for P3, P8, P10, and P11. Phage Lambda DNA was treated with *Eco*RV to serve as a positive control and undigested Lambda phage DNA served as a negative control.
