# Supplementary material for: Bacteriophages specific to Shiga toxin-producing Escherichia coli exist in goat feces and associated environments on an organic produce farm in Northern California, USA
Source: PLoS One. 2020 Jun 11;15(6):e0234438. doi: 10.1371/journal.pone.0234438 (PMC7289414; doi:10.1371/journal.pone.0234438)
Supplement: S1 Table — Strains excluding O157-2 were obtained from USDA ARS WRRC. Strain O157-2 was obtained from the ATCC, American Type Culture Collection, Manassas, VA. Strain O121-1 was obtained from the CDC, Centers for Disease Control and Prevention, Atlanta, GA. (DOCX) [file pone.0234438.s003.docx]

**Table S1.** **STEC strains used for isolating STEC-specific phages**. Strains excluding O157-2 were obtained from USDA ARS WRRC. Strain O157-2 was obtained from the ATCC, American Type Culture Collection, Manassas, VA. Strain O121-1 was obtained from the CDC, Centers for Disease Control and Prevention, Atlanta, GA.

|  | | | **Virulence features** | | | | |
| --- | --- | --- | --- | --- | --- | --- | --- |
| **Strain** | **ID** | **Source** | **O type** | **H type** | ***stx*1** | ***stx*2** | ***eae*** |
|  |  |  |  |  |  |  |  |
| O103-1 | RM13322 | cattle feces | 103 | 2 | + | - | + |
| O103-2 | RM10744 | cattle feces | 103 | - | + | - | + |
| O111-1 | RM11765 | water | 111 | - | + | - | + |
| O111-2 | RM14488 | water | 111 | - | + | + | + |
| O121-1 | 96-1585 | CDC | 121 | 19 | - | + | + |
| O121-2 | RM8082 | cattle feces | 121 | - | + | - | - |
| O145-1 | RM10808 | cattle feces | 145 | - | + | - | + |
| O145-2 | RM9872 | cattle feces | 145 | - | - | + | - |
| O157-1 | RM18959 | water | 157 | 7 | + | + | + |
| O157-2 | ATCC 35150 | human feces | 157 | 7 | + | + | + |
| O26-1 | RM18132 | water | 26 | - | + | - | + |
| O26-2 | RM17133 | water | 26 | - | + | - | - |
| O45-1 | RM10729 | cattle feces | 45 | - | + | - | - |
| O45-2 | RM13752 | cattle feces | 45 | - | + | - | - |
