# Supplementary material for: Bacteriophages specific to Shiga toxin-producing Escherichia coli exist in goat feces and associated environments on an organic produce farm in Northern California, USA
Source: PLoS One. 2020 Jun 11;15(6):e0234438. doi: 10.1371/journal.pone.0234438 (PMC7289414; doi:10.1371/journal.pone.0234438)
Supplement: S2 Table — (DOCX) [file pone.0234438.s004.docx]

| **Table S2. RT-PCR Primers and probes used for *stx* gene screening in phage or bacteria** [34]. | | |
| --- | --- | --- |
|  |  |  |
| **Name** | **Sequence** | **Position** |
| *Stx*1 forward | CATCGCGAGTTGCCAGAAT | 803 |
| *Stx*1 reverse | TCCCACGGACTCTTCCATCT | 874 |
| *Stx*1 probe | Q670-ATCTGATGATTTCCTTCTATGTGTCCG-BHQ2 | 825 |
|  |  |  |
| *Stx*2abc for. | GGACCACATCGGTGTCTGTTATT | 167 |
| *Stx*2abc rev. | CCCTCGTATATCCACAGCAAAAT | 234 |
| *Stx*2abc probe | HEX-CCACACCCCACCGGCAGT-BHQ1 | 192 |
|  |  |  |
| *Stx*2ex for. | GAAACTGCTCCTGTTTATACGATGAC | 616 |
| *Stx*2ex rev. | CCGGAAGCACATTGCTGAT | 697 |
| *Stx*2ex probe | FAM-CCCCCAGTTCAGAGTGAGGTCCACG-BHQ1 | 675 |
| Position is relative to the coding region of *stx*1 and *stx*2A from O157 strain EDL933 | | |
