# Supplementary material for: Bacteriophages specific to Shiga toxin-producing Escherichia coli exist in goat feces and associated environments on an organic produce farm in Northern California, USA
Source: PLoS One. 2020 Jun 11;15(6):e0234438. doi: 10.1371/journal.pone.0234438 (PMC7289414; doi:10.1371/journal.pone.0234438)
Supplement: S3 Table — (DOCX) [file pone.0234438.s005.docx]

| **Table S3. Luminex MagPix Molecular STEC Serotyping primers and probes [35].** | | | |
| --- | --- | --- | --- |
| **O Serogroup** | **Gene** | **Sequence** | **Gene position (bp)** |
| O26 | *wzx* | ttttatctggcgtgctatcg | 557-577 |
|  |  | Biotin-cggggttgctatagactgaa | 784-804* |
|  |  | Uni-link-tggcactcttgcttcgcctg | 720-740 |
|  |  |  |  |
| O45 | *wzy* | tacgatttcacaagcttcca | 769-789 |
|  |  | Biotin-tgcaatcgcataaggaaata | 1003-1023* |
|  |  | Uni-link-tcgcgggctcccttattgtg | 917-937 |
|  |  |  |  |
| O91 | *wzx* | catgctgctcattcttctca | 266-286 |
|  |  | Biotin-tggagtttgcaacaaacaaa | 380-400* |
|  |  | Uni-link-aaatggtttgctgcgacgct | 358-378 |
|  |  |  |  |
| O103 | *wzx* | gggcttgtattgttgtaccg | 896-916 |
|  |  | Biotin-agtggcaaacagccaactac | 1045-1065* |
|  |  | Uni-link-tcggggattttctgcggatt | 1025-1045 |
|  |  |  |  |
| O104 | *wzx* | tgcgggattaatatcctttg | 591-611 |
|  |  | Biotin-acgccctagaaacctgactt | 854-874* |
|  |  | Uni-link-cgcaggttttattgtcgcgc | 780-800 |
|  |  |  |  |
| O111 | *wzx* | caatccaatttgcatcttca | 75-95 |
|  |  | Biotin-accgcaaatgcgataataac | 294-314* |
|  |  | Uni-link-tggaggatgttccgcatgga | 189-209 |
|  |  |  |  |
| O113 | *wzx* | tgaccttacttcctgcgaat | 752-772 |
|  |  | Biotin-agcaccacgataggattgaa | 977-997* |
|  |  | Uni-link-cctgggaggaggctgcaaaa | 953-973 |
|  |  |  |  |
| O121 | *wzy* | tggatggcattcctcagtat | 809-829 |
|  |  | Biotin-agcaagccaaaacactcaac | 1043-1063* |
|  |  | Uni-link-ttaacacgggcgtggttgga | 920-940 |
|  |  |  |  |
| O128 | *wzx* | tcgatcgtcttgttcaggtt | 1123-1143 |
|  |  | Biotin-gaatgcaatgggcaattaac | 1298-1318* |
|  |  | Uni-link-gggttgcacaattggcctcc | 1184-1204 |
|  |  |  |  |
| O145 | *wzy* | tgttcctgtctgttgcttca | 224-244 |
|  |  | Biotin-atcgctgaataagcaccact | 495-515* |
|  |  | Uni-link-tgggctgccactgatgggat | 442-462 |
|  |  |  |  |
| O157 | *wzx* | ataatccagccagcaaagtg | 1026-1046 |
|  |  | Biotin-ggtgctgctctgacattttt 1 | 1141-1161* |
|  |  | Uni-link-gcccaccactaatttgccga | 1047-1067 |
|  |  |  |  |
| n/a | *eae* | tggaacggcagaggttaatc | 630-650 |
|  |  | Biotin-gtaaagcgggagtcaatgta | 747-767* |
|  |  | Uni-link-tgctggcatttggtcaggtc | 719-739 |
|  |  |  |  |
| n/a | *aggR* | ttaagacgcctaaaggatgc | 1448-1468 |
|  |  | Biotin-acagaatcgtcagcatcagc | 1540-1560* |
|  |  | Uni-link-agatgcttgcagttgtccga | 1498-1518 |
| *Reverse complement | | |  |
