# Supplementary material for: Bacteriophages specific to Shiga toxin-producing Escherichia coli exist in goat feces and associated environments on an organic produce farm in Northern California, USA
Source: PLoS One. 2020 Jun 11;15(6):e0234438. doi: 10.1371/journal.pone.0234438 (PMC7289414; doi:10.1371/journal.pone.0234438)
Supplement: S4 Table — The Luminex MagPix CCD camera interrogates each bead and detects the amount of reporter/fluorescence bound to each bead region then calculates the mean of the fluorescence reads for that particular bead region. (DOCX) [file pone.0234438.s006.docx]

| **Table S4. Luminex MagPix Mean Fluorescent Intensity (MFI) Results of Soil-Isolated STEC**. The Luminex MagPix CCD camera interrogates each bead and detects the amount of reporter/fluorescence bound to each bead region then calculates the mean of the fluorescence reads for that particular bead region. | | | | | |
| --- | --- | --- | --- | --- | --- |
|  | **Mean MFI at each bead region** | | | | |
| **Bead Region** | **nfH2O** | **(+) Control 1** | **(+) Control 2** | **(+) Control 3** | **Soil-isolated STEC** |
| *eae* | 311.7 | 3160.2 | 2682 | 2966 | 229.3 |
| *agg*R | 96 | 109.5 | 103.5 | 105.7 | 106.7 |
| O157 | 100.8 | 1710.7 | 103.8 | 100.8 | 109 |
| O104 | 903.7 | 565.8 | 329.7 | 331.7 | 251 |
| O111 | 94.7 | 122.5 | 380.8 | 101.2 | 102.5 |
| O113 | 106.7 | 120 | 112.3 | 112.8 | 114.8 |
| O128 | 98.3 | 106 | 104 | 106.7 | 104.7 |
| O145 | 92.7 | 1255 | 95.7 | 99.8 | 97.5 |
| O26 | 93.8 | 139.7 | 151.5 | 248.7 | 101 |
| O45 | 91.7 | 99.2 | 98.3 | 149.8 | 96.7 |
| O91 | 105 | 108.3 | 102 | 104.8 | 102 |
| O103 | 109.3 | 1737 | 107 | 108.5 | 108.2 |
| O121 | 103.7 | 1201.8 | 105 | 109.3 | 109.7 |
|  | | | | | |
