# Supplementary material for: Bacteriophages specific to Shiga toxin-producing Escherichia coli exist in goat feces and associated environments on an organic produce farm in Northern California, USA
Source: PLoS One. 2020 Jun 11;15(6):e0234438. doi: 10.1371/journal.pone.0234438 (PMC7289414; doi:10.1371/journal.pone.0234438)
Supplement: S5 Table — For data analysis, the S/N ratio for each analyte, or amplified DNA of presumptive isolates bound to a specific region of the MagPlex bead and bearing a reporter, was calculated against the background noise by dividing the mean fluorescent intensity (MFI) of the analyte by the MFI of the nuclease-free H2O sample. Analytes with a signal-to-noise ratio of >5.0 were considered to be positive for that analyte, however, due to the sensitive nature of the assay, any analytes resulting in lone signals of >1 for only one serogroup were subjected to serotype confirmation. (DOCX) [file pone.0234438.s007.docx]

| **Table S5. Luminex MagPix Signal-to-Noise (S/N) Ratios of Soil-Isolated STEC**. For data analysis, the S/N ratio for each analyte, or amplified DNA of presumptive isolates bound to a specific region of the MagPlex bead and bearing a reporter, was calculated against the background noise by dividing the mean fluorescent intensity (MFI) of the analyte by the MFI of the nuclease-free H2O sample. Analytes with a signal-to-noise ratio of >5.0 were considered to be positive for that analyte, however, due to the sensitive nature of the assay, any analytes resulting in lone signals of >1 for only one serogroup were subjected to serotype confirmation. | | | | | |
| --- | --- | --- | --- | --- | --- |
|  | **Signal-to-noise ratio at each bead region** | | | | |
| **Bead Region** | **nfH2O** | **(+) Control 1** | **(+) Control 2** | **(+) Control 3** | **Soil-isolated STEC** |
| *eae* | 1 | 10 | 9 | 10 | 1 |
| *agg*R | 1 | 1 | 1 | 1 | 1 |
| O157 | 1 | 17 | 1 | 1 | 1 |
| O104 | 1 | 1 | 0 | 0 | 0 |
| O111 | 1 | 1 | 4 | 1 | 1 |
| O113 | 1 | 1 | 1 | 1 | 1 |
| O128 | 1 | 1 | 1 | 1 | 1 |
| O145 | 1 | 14 | 1 | 1 | 1 |
| O26 | 1 | 1 | 2 | 3 | 1 |
| O45 | 1 | 1 | 1 | 2 | 1 |
| O91 | 1 | 1 | 1 | 1 | 1 |
| O103 | 1 | 16 | 1 | 1 | 1 |
| O121 | 1 | 12 | 1 | 1 | 1 |
